# Developing a Comparative Docking Protocol for the Prediction of Peptide Selectivity Proﬁles: Investigation of Potassium Channel Toxins

**DOI:** 10.3390/toxins4020110

**Published:** 2012-02-06

**Authors:** Po-Chia Chen, Serdar Kuyucak

**Affiliations:** School of Physics, Building A28, University of Sydney, NSW 2006, Australia; Email: poker@physics.usyd.edu.au

**Keywords:** protein-protein docking, scorpion toxins, K_v_1.1, K_v_1.2, K_v_1.3, selectivity, α-KTx, HADDOCK, comparative docking

## Abstract

During the development of selective peptides against highly homologous targets, a reliable tool is sought that can predict information on both mechanisms of binding and relative afﬁnities. These tools must ﬁrst be tested on known proﬁles before application on novel therapeutic candidates. We therefore present a comparative docking protocol in HADDOCK using critical motifs, and use it to “predict” the various selectivity proﬁles of several major αKTX scorpion toxin families *versus *K_v_1.1, K_v_1.2 and K_v_1.3. By correlating results across toxins of similar proﬁles, a comprehensive set of functional residues can be identiﬁed. Reasonable models of channel-toxin interactions can be then drawn that are consistent with known afﬁnity and mutagenesis. Without biological information on the interaction, HADDOCK reproduces mechanisms underlying the universal binding of αKTX-2 toxins, and K_v_1.3 selectivity of αKTX-3 toxins. The addition of constraints encouraging the critical lysine insertion conﬁrms these ﬁndings, and gives analogous explanations for other families, including models of partial pore-block in αKTX-6. While qualitatively informative, the HADDOCK scoring function is not yet sufﬁcient for accurate afﬁnity-ranking. False minima in low-afﬁnity complexes often resemble true binding in high-afﬁnity complexes, despite steric/conformational penalties apparent from visual inspection. This contamination signiﬁcantly complicates energetic analysis, although it is usually possible to obtain correct ranking via careful interpretation of binding-well characteristics and elimination of false positives. Aside from adaptations to the broader potassium channel family, we suggest that this strategy of comparative docking can be extended to other channels of interest with known structure, especially in cases where a critical motif exists to improve docking effectiveness.

## 1. Introduction

The inhibition of potassium channels by peptides from animal venoms is a subject of broad interest for its physiological and therapeutic applications [1]. The distribution of channels being ubiquitous and varied, efﬁcient and safe targetting relies upon the selective binding of pharmacological agents to speciﬁc subtypes involved. These include K_v_1.3 and K_Ca_3.1 in T-cell mediated diseases [2,3,4], K_v_1.4 and 4.x in neuropathic pain [5,6], and others.

There are many possible sources of channel-blocking peptides, including snakes [7], spiders [8], scorpions [9], sea-anemones [10] and cone-snails [11], of which the most diverse collection currently recorded are the αKTX scorpion toxins [12] that target voltage-gated potassium channels (K_v_). The similarity of basic functional motifs across the various folding patterns is remarkable, indicating a strong association with the particular characteristics of the channel surface. Moreover, these toxins also offer a wide-range of afﬁnities against individual sub-types—a characteristic that underlies current efforts to design pharmaceutically useful toxins.

The selectivity proﬁle of a given toxin is the primary property of interest in pharmaceutical design. This has generally been studied in the context of K_v_ and K_Ca_ selectivity [13,14,15,16] as well as K_v_1 subtype selectivity. It is of great interest to construct a methodology that will assist in the prediction of a toxin’s selectivity proﬁles, given its sequence and structure. This task can be ﬁlled by protein-protein docking, which serves as a tool for the prediction and validation of functional residues in association with mutagenesis studies. Studies involving docking combination with experiment include charybdotoxin (ChTX) [17], iberiotoxin IbTX [16], Css20 [18], agitoxin-2 (AgTX2) [19], maurotoxin (MTX) and Pi1 [20], ADWX-1 [21], cobatoxin-1 (coba-1) [22], and many others. The result from these various works is an agreed set of motifs that determine general K_v_-binding [1,9], although the exact motifs governing subtype selectivity have not been fully elucidated.

Protein-protein docking programs [23] are numerous [24,25,26,27], and are being continually improved by the community and tested in open avenues such as CAPRI [28]. The primary goal of these programs is the correct prediction of bound complexes from the apo-conformations of its constituents. Success currently depends on the representation of molecular ﬂexibility, whether in the input-conformation(s) [29,30] or the degree to which protocols can undertake the ﬂexible transformations required to move apo-states to holo-states [31,32]. Meanwhile, afﬁnity predictions from structure [33] are usually the domain of more accurate formulations such as MM/PBSA and molecular dynamics [34]. The equivalent task in docking programs have been explored for small molecule binding [35,36,37], but relatively few studies have been carried out at the protein-protein level.

In this work, we seek a uniﬁed protocol that can be applied to arbitrary toxin-channel pairs. This report is a proof-of-principle application of the HADDOCK software for such purposes, tested against the family of αKTX-scorpion toxins. We seek to show by numerous examples the extent of information that can be obtained by comparative docking: signiﬁcant contact-pairs, residue locations that exert selectivity, and whether in-vivo afﬁnity can be predicted. The rat/mouse K_v_ 1.1, 1.2 and 1.3 channel have been chosen as targets because of the comprehensive afﬁnity data available across the families tested. We draw upon docking trials of over 25 αKTx scorpion toxins against these channels, and three conotoxins as control (see Table 1). Detailed methodology is provided in the hopes that the principles of this methodology can be broadened to other channels of interest.

## 2. Methodology

All docking and MD simulations have been carried out with HADDOCK [25,38] and NAMD [39], respectively. Visualisations of molecules and plots are in VMD [40], gnuplot, and xmgrace.

### 2.1. Conformer Preparations

Toxin coordinates listed in Table 1 have been assembled from the Protein Data Bank. MD-ensembles have been generated for docking input, using the ﬁrst conformer from NMR-ensembles in 11-ns MD simulations in NAMD under constant pressure and temperature (NPT)-conditions with 150 mM KCl. Where docking using NMR-ensembles generate reasonable docking complexes, NMR-ensembles have been used. Simulation-boxes for each equilibration is based on molecular volume followed by padding with water of 10 Å in each axis, resulting in ∼ 7000-atom systems. Frames were collected every 250 ps over the last 8 ns, yielding 32 conformations.

Previous studies have identiﬁed afﬁnity changes of ∼3-fold with respect to C-terminal amidation [41,42]. We have veriﬁed where possible the correct amidation states for toxins used in experimental studies—but have otherwise assumed carboxylate forms for all synthetic sources.For example, the structure of natively isolated noxiustoxin (NTX) is C-terminal amidated [43]. However, this was removed in line with afﬁnity studies [44] that used a synthetic source [45] without amidation.

Channel coordinates for K_v_1.1 and K_v_1.3 have been prepared by taking the crystal structure of K_v_1.2-paddle chimera (2R9R.pdb [46]) and mutating necessary residues via the Mutator plug-in from VMD. All histidines have been set as neutral, and protonated at N_є_. The mutated channels have been equilibrated in POPE-membrane under step-wise decreasing backbone and side-chain constraints, taking place over ∼3 ns. Conformers were then collected in 200-ps intervals over a 6 ns unrestrained simulation giving 31 conformations.

**Table 1:** List of peptide toxins included in docking studies, removing members for which experimental information is unavailable. Entries restrictedto current-block studies of rat K_v_s on Xenopus oocytes unless otherwise stated. Starred entries next to K_v_1.3-afﬁnity data have been derived fromhuman channels, and stars at the end of amino acid sequences indicate C-terminal amidation. Amino-acids Z and O indicate 5-oxoproline and 4-hydroxyproline, respectively, and have been parametrised manually for docking and molecular dynamics. The putative pore-Lys is bolded ineach sequence.

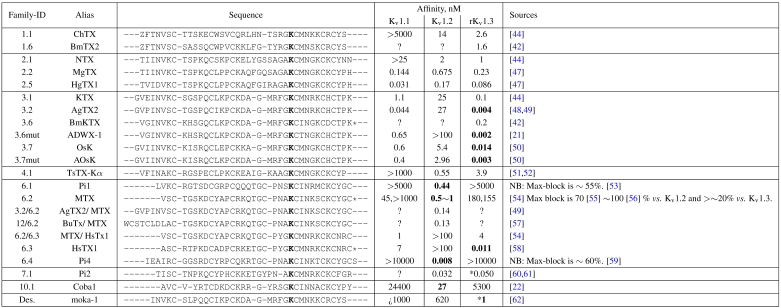

### 2.2. Methods of Analysis

In an effort to apply our methodology across the broadest subset of toxins, we have used characteristics common to αKTx toxins as parameters to analyse docking results: (1) a side-chain lysine that inserts into the ion-conducting pore (pore-Lys), and (2) the *β*-sheet covering residues between 2-upstream and 9-downstream of pore-Lys that must be in contact for this insertion to occur. As pore-Lys insertion is a common characteristic amongst αKTx toxins [9,63], we deﬁne a distance between its amine and the centre of the plane formed by tyrosine carbonyls in the selectivity ﬁlter (TVGYG). The length and strand-turn-strand secondary structure of the *β*-sheet is also conserved in all of the αKTx toxins studied, along with locations of hitherto known functional residues. This forms a suitable common RMSD-reference for toxins and removes the performance bias associated with top-complex RMSDs in individual docking trials. The reference coordinates have been taken from the NMR-based structure of KcsAmut with bound ChTX [64], and we will refer to this measure as the “*β*-sheet Cα RMSD” for the rest of this report.

### 2.3. Docking Protocols

We construct two general scenarios under which a docking program is likely to be used: blind-docking and binding site reﬁnement. The former takes place when binding site information is generally *unknown*, and docking is used as a source of inspiration for further experimental validation, while the latter is a converse scenario where some information is *known *(usually through partial mutagenesis data) and docking is used to visualise this and provide further insights to the interaction. In blind trials, 20,000 initial rigid body complexes are generated with randomised ambiguous interaction restraints (AIRs) determined by HADDOCK. Conformers are selected systematically in order to represent all possible pairings. The top-200 initial encounter-complexes by HADDOCK-energy are then reﬁned in simulated-annealing, split into sidechain-ﬂexible stages and all-atom ﬂexible stages in explicit solvent. Re-ranking of these reﬁnements give the results of docking.

In scenarios of binding-site reﬁnement, we have assumed prior knowledge of the lysine-insertion motif and deﬁned a set of constraints and additional considerations to aid in docking. A set of 3 Å-distance restraints is created using pore-Lys as the centre of the interaction surface, with reference to the terminology of “active” and “passive” residues as deﬁned by HADDOCK. Active residues (*i.e.*, residues with explicit restraints) span pore-Lys and two additional residues nearby (2-upstream and 2-downstream along the *β*-sheet). Although pore-Lys forms hydrogen-bonds with the tyrosine carbonyls separating the S0/S1 ion binding-sites in the ﬁlter, this residue is largely buried and cannot act as an effective restraint. Therefore, we include glycines at the end of the selectivity ﬁlter as active residues. Passive residues (potential partners of restraints) include all physically-adjacent residues of active residues.

In terms of test and production runs, 5000 initial rigid-body complexes are generated and the top-200 selected for further reﬁnement as above. Channel symmetry has been included in docking after spurious observations of misalignment between monomers—these constraints consist of four C2-symmetry pairs binding opposing P-helices and the top half of S6-helices, separately. C4-symmetry has not been implemented in HADDOCK at the time of testing. Ion and water occupancy at crystallographic coordinates were also considered, assuming the canonical high conductivity states of S0/S2/S4 or S1/S3, then removing waters/ions at S0 and S1 to facilitate pore-Lys insertion. This includes crystallographic waters found behind the selectivity ﬁlter. Docking runs utilising all of the above considerations will be described as “optimal” in this study.

## 3. Results and Discussion

### 3.1. Channel Morphology

**Figure 1:**
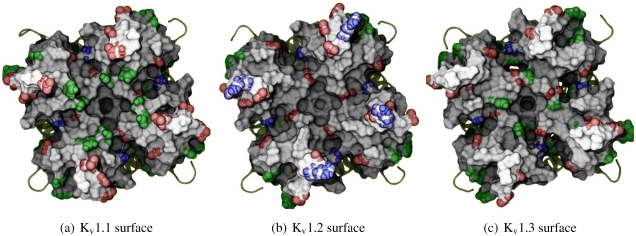
Exterior view of the K_v_1 channels, highlighting the differences in morphology and residue types. Sidechains of exposed basic (blue), acidic (red) and aromatic residues (green) have been given coloured spheres to indicate the nature of potential interactions. Surface calculated by MSMS at 3 Å^−2^ density and 1.5 Å probe radius and rendered with ambient occlusion by Tachyon in VMD before post-processing.

Before exploring the details of toxin-channel interactions, it is important to remind ourselves of the unique characteristics of the channel surface. To give a spatial understanding of this, we show an MD-snapshot of the three Kv1-channels in Figure 1 and list their sequences in Table 2. All residue numberings will be according to K_v_1.2 for consistency. The most critical characteristic to note is the relative size of turret residues at positions 353–359, and in particular 357 and 381 most proximal to the pore. K_v_1.3 presents a broad and shallow surface for toxin-binding (G357 and H381), while K_v_1.1’s vestibule is signiﬁcantly narrowed by bulky residues H357 and Y381. We note that the role of external space in selectivity has been previously examined between K_v_1.3 and K_Ca_1.1 channels [15], and will also apply such considerations to this study.

It should also be noted that the net charge of the entire exterior is either -20 (K_v_1.1), or -16 (K_v_1.2 and K_v_1.3), taking into account that the pKa of histidines are not signiﬁcantly affected according to propKa calculations [65]. We therefore expect to ﬁnd non-speciﬁc binding by most toxins due to their net positive charge.

**Table 2:** Sequence extract of K_v_1-channels used in this work from the top-half of TM5, pore-helix, selectivity ﬁlter, to the top-half of TM6. The residue numbering have been matched to rat K_v_1.2 (*i.e.*, 2A79.pdb).

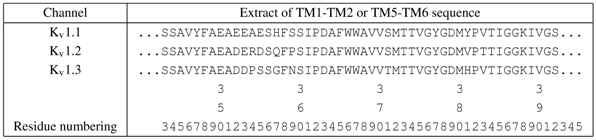

### 3.2. Complex Selection and Ordering of Results

In order to minimise bias in the validation, we devised a set of general criteria that was used to select the complexes shown in this report. Likewise, trends of afﬁnity and experimental mutagenesis studies have been purposefully excluded (as much as we are capable thereof). The conditions for complex selection are thus:

(1) That the complex is correctly assembled. The channel assembly are sometimes distorted in order to satisfy docking constraints, whether in random blind-trials or explicitly given. The program limitations prevent a proper modelling of the membrane environment, which translates into dilation of the K_v_-monomers and destruction of selectivity ﬁlter sites. We judge this perturbation via the separation of opposing monomers and the positions of crystallographic ion and waters in the ﬁlter, and prefer complexes with minimal or no perturbations.

(2) That the secondary/tertiary structures are not signiﬁcantly distorted. Across the entire dataset, we observed a similar tendency for complexes to maximise contact interfaces at a sacriﬁce of conformational integrity. This often appears as a partial loss of toxin secondary structure associated with very deep interactions of sidechain moieties. We believe that this is a by-product of HADDOCK design philosophy that maximises effectiveness of the binding-site search, and will show later in this report that such tendencies can lead to false positives where toxins distort themselves in order to ﬁt into binding modes otherwise not possible.

(3a) That the chosen complex be a member of a cluster, and where multiple clusters are available, the largest and energetically-favoured cluster. The essence of docking being stochastic, repeatability of results is a primary conﬁrmation of success. This criterion should excludes isolated outliers that appear at signiﬁcantly lower energies than the main cluster.

(3b) That the chosen complex be of a low energy member of its cluster, and representative of the overall interactions observed within the cluster. The docking protocol implemented rarely optimises every aspect of the interaction in question, and many complexes exhibit subsets of the interactions that are possible for the binding mode in question. We choose one that shows best the characteristics of the cluster.

Due to the large number of toxins and the similarity of observations within several families, we will group these discussions by αKTx family IDs and sequence homology. As the family-1 toxins are generally stronger binders to K_Ca_-channels, we will not discuss them in this report. We will instead begin with the αKTx-2 family that is generally effective against all three K_v_-subtypes.

### 3.3. αKTx-2

**Figure 2:**
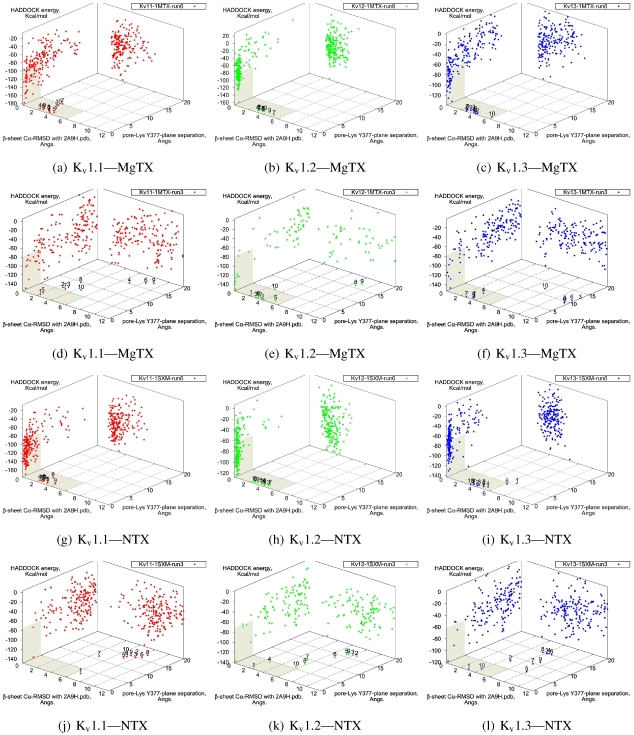
Comparison between constrained-and blind-docking data for two members of αKTX-2, margatoxin (MgTX, 1MTX.pdb) and noxioustoxin (NTX, 1SXM.pdb). Constrained docking results are presented in the 1st and 3rd rows, while blind docking results are in rows 2 and 4. Colours red, green and blue correspond to K_v_1.1, K_v_1.2, and K_v_1.3 complexes, respectively. Numbers 1–10 above the dots indicate the rank of the complex at that conﬁguration.

The tested family-2 toxins comprise three toxins: noxiustoxin (NTX, αKTx-2.1), margatoxin (MgTX, 2.2), and hongotoxin (HgTX1, 2.5). The latter two bind to tested Kv-channels at pM afﬁnity, while NTX bind at nM afﬁnity and does not interact strongly with K_v_1.1. We will show the results of blind and constrained trials for NTX and MgTX in Figure 2. The docking performances of HgTX1 resemble that of MgTX and are therefore not shown here.

The general performance of HADDOCK is visually represented by sorting complexes according to pore-Lys insertion, *β*-sheet RMSD and HADDOCK-energy. Potential native complexes are therefore near the bottom-left border, which show low separation of pore-Lys from the pore. MgTX constrained-dockings (Figure 2a–c) show visible clustering of complexes near the expected result—the top-ten complexes are relatively close and surrounded by a funnel in the energy surface, although they are not always members of the same cluster. When supported by contact analysis, these observations suggest that the toxin is a good binder.

The net effect of constraints is to heavily concentrate searches around a putative binding orientation. Although sparsely populated, similar conﬁgurations are found between the best blind and best constrained complexes. It is interesting to note that blind results (*i.e.*, without search bias) for NTX results yield fewer canonical binding than picomolar binders MgTX and HgTX2, and moreover, none of the top-complexes *vs.* K_v_1.1 are canonical (compare Figure 1(i) with 1(l)). This is suggestive of some correlation between afﬁnity and docking success as measured by hit frequency. It also implies that constraints may force ligands to bind where it should not—an assumption that will need to be tested for novel toxins. We will return to this topic when discussing potential afﬁnity predictions. For now, we also note that conformational distortions are more common in K_v_1.1 complexes, particularly for the forced NTX example. This is evidence of a possible false-positive, and we will conﬁrm this with more examples in other families.

**Figure 3:**
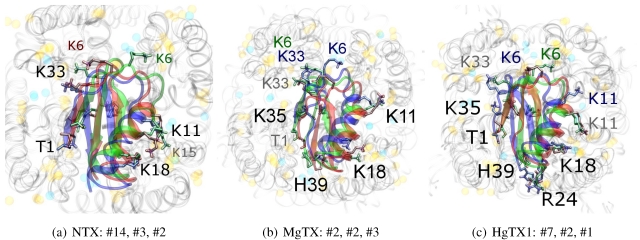
Superimposed chosen Kv-docked complexes of the family-2 toxins from constrained results. Colours red, green and blue correspond to K_v_1.1, K_v_1.2, and K_v_1.3 complexes, respectively. Labelled residues indicate charged elements that interact (< 5 Å) with the channel in either all three cases (black) or two out of three cases (grey). Isolated charge contacts are labelled by colour of complex. Numbers after labels indicate the actual complex selected as sorted by HADDOCK energy, in the same order. Other interacting residues have been left unlabelled for simplicity.

The complexes *vs.* all three channels in this family reside at similar *β*-sheet RMSD. This is because the modes are super-imposable (Figure 3). MgTX and HgTX1 share a common binding mode that emphasises contact with conserved residues E/D353, D363 and D379 via basic-ring residues such as K11, K35 and H38 (likely charged due to its proximity with several carboxylic moieties). The addition of R24 in HgTX1 increases the net afﬁnity relative to MgTX, from its additional salt-bridge without signiﬁcantly perturbing the binding mode.

Are there any other residues that deﬁne the characteristics of family-2? The contacts of the *β*-sheet are small up-stream of pore-Lys K28 (SAGAK), and large downstream (KCMNGKCKCYPH). This combination forms a relatively ﬂat interface that encourages a centralised location over the conducting pore. Such a position brings maximum weight to the inﬂuence of position 381—where K_v_1.1’s Y381 should constitute the greatest source of perturbation—and decreases the contribution of turret residues to selectivity. This reasoning applies in particular to NTX, whose helices lack the double proline P15/P16 that allows MgTX and HgTX1 helices to bend and accommodate Y381. The destabilisation of the common binding mode is most evident in the inability of K_v_1.1-NTX blind-trials to identify this mode, and the signiﬁcantly increased frequency of distortion in results. NTX also possesses the mutation H39N, which is a loss of charge that may be the explanation for a decreased afﬁnity relative to MgTX and HgTX1.

### 3.4. αKTx-4

**Figure 4:**
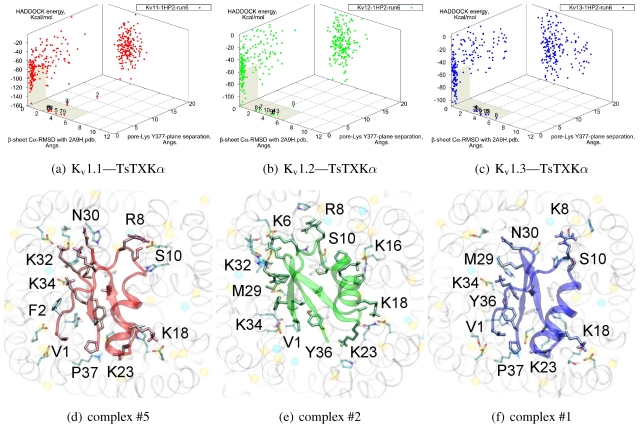
Constrained-docking results for αKTx-4 member tityustoxin-K α *vs.* K_v_1.1 (red), K_v_1.2 (green) and K_v_1.3 (blue). (a–c) HADDOCK Energy plots against pore-Lys distance and *β*-sheet RMSD with 2A9H.pdb. (d–f) Selected complexes are shown as a visual aid to docked conformations.

We will now turn to family-4, whose *β*-sheet similarity is closest to family-2. Tityustoxin-K α (TsTXKα, 4.1) exhibits a weak K_v_1.2 preference over K_v_1.3, but like NTX it does not bind K_v_1.1. Docking for this toxin reveals two competing modes at ∼2.5 and ∼4.5 Å RMSD for both K_v_ 1.2 and K_v_1.3 (Figure 4). Although we choose to present a K_v_ 1.2 complex from the ∼4.5 Å cluster based on cluster size and frequency, complex #1 conformations (∼2.5 Å) are very similar for both channels. It is important to note that we again observe analogous binding modes *vs.* K_v_1.1, despite the lack of experimental afﬁnity. The complex shown (#5) for this channel is a marginally-acceptable model, intended to illustrate the kind of toxin distortions that may be found for conﬂicting pairings.

The presence of two modes appears to be a product of increased symmetry and toxin size. The 2.5 Åmode favoured by K_v_1.3 emphasises charge interactions with D379s by K23 and K34, and an extended toxin-conformation with the α-helix pulled closer to the channel exterior. As a whole, contact pairings of this mode are similar to the modes found for αKTx-2 toxins. On the other hand, the 4.5 Å mode favoured by K_v_1.2 emphasises basic-ring interactions with turret charges. TsTXKα’s shorter *β*-sheet and smaller constituent amino-acids permits this rotated mode, where we observe mirror pairs such as S10/Y36, V1/K16 and K18/K32 that interact with equivalent residues on two opposing channel monomers. The two modes found by HADDOCK are thus related by a rotation of 30–45 degrees along the channel axis in connection with the relative displacement of these residues.

The potential existence of multiple modes becomes more reasonable if we study the symmetry properties of the interaction. The basic-ring distributions of many toxins mimic the channel’s tetrameric nature. Likewise, H-bonding partners such as S10 and Y36 form opposing pairs that bind to backbone carboxyls. On the channel, position 355 is marginally farther from the pore than D379, which means the inclusion of a negative charge here increases the charge symmetry to 8-fold. Where permitted by the toxin’s geometry (*i.e.*, a favourable match with residue 381) this favours modes where the toxin is capable of exploiting charges on the turret, whereas its replacement S355 in K_v_1.3 offers only H-bonding stabilisation.

Given the accuracy limits of docking, the small separation in energy of the two clusters is probably not sufﬁcient evidence to conﬁdently assert that TsTXKα does bind in different orientations to the two channels. The above analysis can be conﬁrmed by mutagenesis of residues that preferentially affect one mode over the other, such as G9 and K23 to reduce K_v_1.3 binding, or an insertion of a *β*-sheet residue to destabilise the rotated K_v_1.2 mode.

### 3.5. αKTx-3

The tested αKTx-3 toxins number six: kaliotoxin (KTX, 3.1), Agitoxin-2 (AgTX2, 3.2), BmKTX (3.6), ADWX-1 (3.6_mut_), OsK1 (3.7), and AOsK1 (3.7_mut_). All of these toxins display strongest experimental binding to K_v_1.3 channels, of which ADWX-1 and AOsK1 are rationally designed mutants and have putatively improved selectivities or afﬁnity for K_v_1.3. We will focus our discussion on ADWX-1, AOsK1, and AgTX2 for the sake of brevity and state simply that the other three toxins show similar docking performance and binding conﬁgurations.

**Figure 5:**
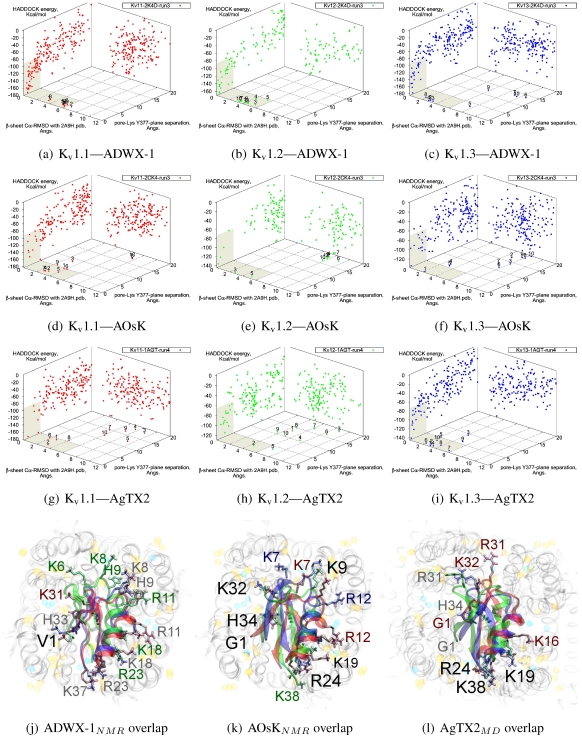
Blind docking data for three family-3 toxins. Colours red, green and blue correspond to K_v_1.1, K_v_1.2, and K_v_1.3 complexes, respectively. Subscripts of the toxin show the source input ensembles used in docking. (a–i) Energy, RMSD and lysine insertion for toxin and channel pairs; (j–l) Superimposed images of chosen complex according to lowest HADDOCK energy with correct lysine-insertion. Labelled residues indicate charged elements that interact with the channel in all three cases (< 5 Å), although not necessarily at the same location. Pore-inserting lysine is unlabelled for simplicity.

As these toxins are generally low-picomolar binders to K_v_1.3, we tested whether blind-docking is sufﬁcient to isolate putative binding orientations (Figure 5). Docking performance is generally acceptable and ranks complexes with canonical pore-Lys insertion well. Where found, the majority of canonical complexes *vs.* K_v_1.3 and some complexes *vs.* K_v_1.1 share the interaction of R23/24(−3) with the channel cleft near D363, in addition to pore-Lys. This feature is conﬁrmed in all constrained-dockings with clustering around similar complexes (shown for ADWX-1 in Figure 7 later). AOsK1 docking with NMR-conformations appears to also bind with the *N*-terminal proximal to the pore, as indicated by a distant cluster on the energy surface. The same phenomenon is also observed in AgTX2 docking with NMR conformations (not shown) but reduces when MD-equilibrated conformers are used. These observations lead us to believe that biases in the initial-conﬁguration can lead to large differences in blind-docking. (We discuss such biases in another submitted publication.) Here, we will take the canonical complexes from blind-docking for further analysis.

Compared with the αKTx-2 MgTX, the αKTx-3 binding site is shifted away from the pore towards the cleft (downwards in diagram). We suggest this is the net effect of the changes on the *β*-sheet (MRFGK). The transfer of the dyad aromatic Y36(+9) to F25(−2) and addition of M(−4) and R(−3) creates a ridge on the upstream *β*-strand that is more suited to the cleft between turrets. R(−3) and charges at the C-terminal constructs an ionic network with D363, D379 and K388(A structural zwitterionic lipid is also located here and participates in the network, although we did not include it in docking). These features lock the toxin to the location shown in Figure 5j–l. Notably, the location of F2 is pressed against the channel surface—we suspect that this is one of the primary causes of selectivity. It is co-located with position 381 in AgTX2 and AOsK1, but is shifted counter-clockwise to a groove between ﬁlter residues in ADWX-1. The energetics of two clusters are again not very well separated except for K_v_1.3, which leads to some doubt on the accuracy of exact details. However, constrained docking does conﬁrm that the modes are distinct and energetically favoured for the respective toxins mentioned.

On the other side of the toxin, we observe proximity of residues both upstream of the helix and at the *β*-turn with the channel turrets. In ADWX-1 these are H9, T29 and N30, which appear to exert strong selectivity effects—the K_v_1.2 binding mode shows signiﬁcant shifting in contact pairs. In AgTX2 and AOsK1, H9 and T29 are replaced by G10/I10 and M30, respectively. These differences may explain the non-afﬁnity for K_v_1.2–ADWX-1 binding via the presence of steric clashes and alternation of local interactions. Therefore, mutations at this half of the toxin are likely to alter the net selectivity without destroying the basic characteristics of αKTx-3.

Other basic-ring members are usually associated with turret charges rather than D363/D379, in contrast with family-2 basic-rings. If the general mode found here is correct, then it may be possible to enhance K_v_1.3 preference by replacing some members with polar residues. Using AOsK1 as a template sequence, G1, K9, K19, and K32 binds E/D355 in the four monomers of K_v_1.1 and 1.2, respectively. This equivalent position is S355 in K_v_1.3, which implies that the loss of salt-bridging while preserving H-bonding will disfavour other K_v_-binding without affecting K_v_1.3 binding adversely.

As it is still unclear whether some channel–toxin interactions possess multiple modes, these predictions may be somewhat simplistic. Docking has produced several related minima that share the main R(−3) anchoring, from which we simply choose the one that appears to be most reasonable for the purposes of this study. It is possible that after eliminating one mode through mutation, some afﬁnity is preserved via a less-favoured but unobstructed mode. Such complexities increase the difﬁculty of building mutant-binding correlations, although the relationship between these modes can be investigated with more accurate computational methods.

### 3.6. αKTx-6

The family-6 toxins have been grouped together due to the presence of an extra disulphide bond, although different selectivity patterns have been observed in its members. This extra pair is commonly found as an additional connection between the C-terminal and the helix-sheet loop upstream of pore-Lys (C1C5-C2C6-C3C7-C4C8), with the exception of maurotoxin (MTX, 6.2) whose network becomes C1C5-C2C6-C3C4-C7C8. We will split the following discussion into K_v_1.2-selective peptides (Pi1, Pi4, and MTX) and K_v_1.1/1.3-selective peptides (HsTX1). The various chimeric constructs based on MTX have been tested in blind-docking but not included in this report, as analysis has been hampered by unorthodox folding in the starting structures.

**Figure 6:**
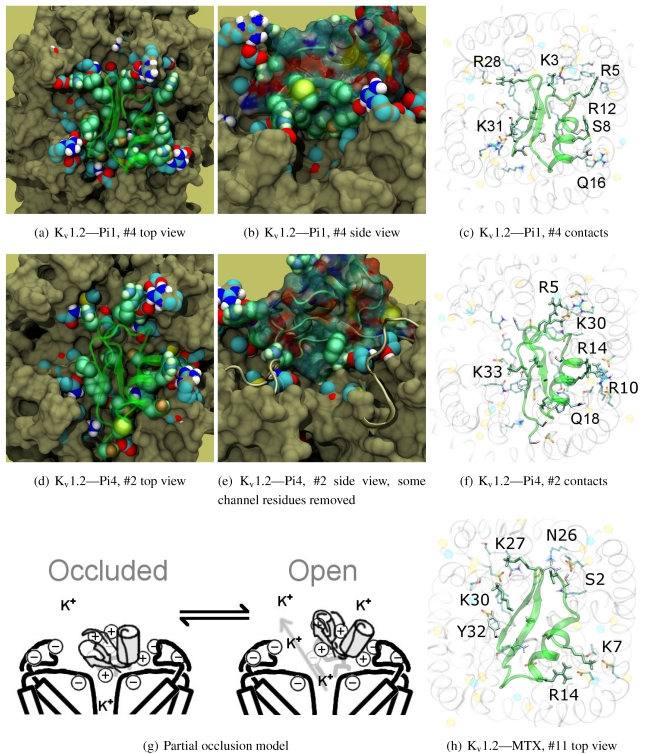
Selected complexes from K_v_1.2-selective members of αKTx-6, providing different views of the interaction. The toxin and channel have been displayed with spheres and surfaces to emphasise the spatial volume of the interaction, and an overview of peripheral contacts is presented. (g) Hypothetical model to explain a maximum block of <100% in this!family.

The majority of peptides in family-6 possess the greatest afﬁnity to K_v_1.2: Pi1 (6.1), MTX, and Pi4 (6.4). It is interesting to note, however, that the maximum current block was shown to be ∼60% at +70 mV depolarisation—indicating some form of partial pore-blocking mechanism. Constrained-docking was carried out for these toxins, which yielded displaced bound positions relative to the pore entrance. The selection of complexes for Pi1 and Pi4 (Figure 6) shows toxins that are tilted towards their helices as a result of the numerous charge contacts there. This results in a partial exposure of the pore vestibule near K30/32(+7) where the inserted pore-Lys is directly visible (Figure 5(b) and 5(e)) Pi1 is stabilised by K3, R5, T7, S8, R12 and Q16 through numerous polar and charged contacts, and Pi4 increases the net afﬁnity through the mutation S8-R10. These are mainly in contact with D355, Q357, and T383 unique to K_v_1.2, in addition to shared moieties such as E/D353. The opposite half on the *β*-sheet is supported only by Y(+9)–N357 and a charge or H-bond contact through K/R(+4). Again, we note that this slanted mode has also been observed in other channels, although very little current block has been observed in experimental studies.

This lop-sided system may explain the partial block observed in current-block studies. It is known that the pore-Lysine site is in competition with extracellular K+ [66,67], and the contacts are relatively weak on the side that partially exposes the ﬁlter. We hypothesise that at positive potentials the weak contacts can break, resulting in a partial unbinding of the toxin and subsequent K+-efﬂux (Figure 5(g)). A maximum block of <100% is explicable by an equilibrium between a bound, pore-occluding mode and a bound, open-pore mode. The numerous contacts along the α-helix spotted in docking provides potential mechanisms where the toxin may remain in close proximity to the pore.

What of MTX? Its experimental data appears to be more complicated. We note that Kharrat *et al.* shows 100% block at depolarisation of 0 mV, while Fajloun *et al.* shows 80% block at depolarisation of 70 mV, both on *Xenopus* oocytes in low exterior K^+^ . This presents us with certain difﬁculties as to which data to use in interpretation. Nevertheless, we ﬁnd a centralised position of MTX compared to the other two toxins. The removal of K3, R5 and R12 and placement of K7 returns the lop-sided nature of contacts elsewhere. K7 and R14 have been conﬁrmed in double mutant cycles [68] to interact with D355, and we observe their direct contacts in Figure 5(h).

**Figure 7:**
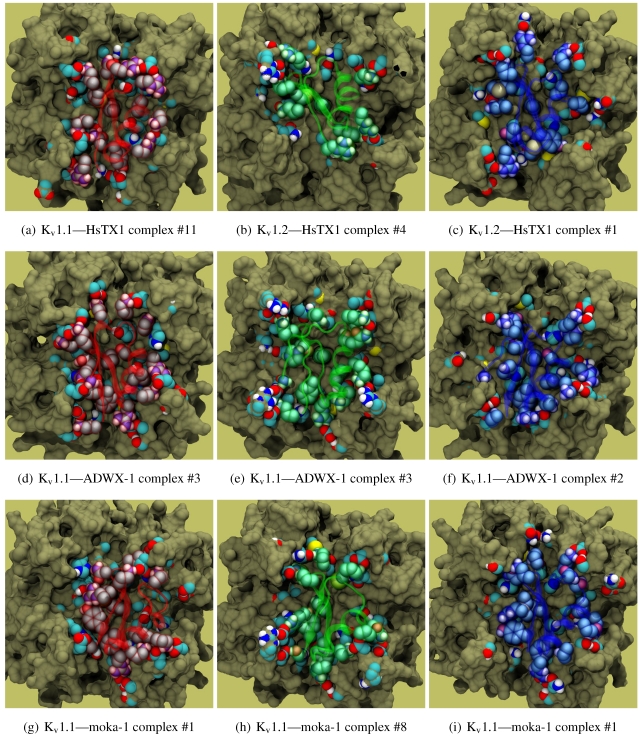
Comparison of docking modes between αKTx-6 HsTX1, αKTx-3 ADWX-1, and combinatorial toxin moka-1 *vs.* K_v_1.1 (red), K_v_1.2 (green) and K_v_1.3 (blue). Toxin residues in contact with the channel have been shown in coloured spheres to visualise the spatial properties in binding.

As noted above, the HsTX1 (6.3) selectivity proﬁle is the opposite of that observed for other members. A complete renovation of the interface is apparent (Figure 7): contacts granted by K28, N32 and R33 ﬁxes the *β*-sheet side of the toxin strongly and places the dyad aromatic Y21(−2) in direct contact with the channel turret of K_v_1.3. We elected to include the binding modes for ADWX-1 and mokatoxin to illustrate the similarities of the K_v_1.3 mode despite the difference in amino-acid sequences. The particular orientation suggests that the mechanisms of selectivity for K_v_1.3 over other channels are broadly similar, and already described for αKTx-3 toxins. The occupancy of residues next to the critical 355 and 357 turret-positions confer local H-bonding as well as surface matching, be it from the asparagine at the (+3)-position [15], Y21(−2) in HsTX1, H9 in ADWX-1, or the double aromatic dyad motif F(−2) and Y(+9) in mokatoxin. It would therefore appear that the same lessons learnt in αKTx-3 binding can apply to these other toxins, and modiﬁcations such as shifting or removing basic ring members not directly beneﬁcial to K_v_1.3-binding will optimise the selectivity-ratio of any similar toxin. Within the bounds of this binding mode, only the charges in its 4-fold symmetry appears to be necessary.

Binding is again observed *vs.* K_v_1.2 where HsTX1 is ineffective up to 100 nM. (A hairline decrease in current is seen in the study proper at 100 nM.) Unlike αKTx-3 toxins where many of the contacts are merely shifted, the position is rotated counterclockwise to a turret-to-turret orientation. Here, HsTX1 loses much of the polar contacts but keeps its charges with new turret pairs. We hypothesise that binding, if present, may be detectable at micromolar concentrations.

### 3.7. Ranking of Toxin Selectivity

HADDOCK is not optimised to predict absolute binding afﬁnities, and its creator (Dr. Bonvin) has previously presented evidence that in the current iteration (HADDOCK2.1) energies are poorly-correlated with experimental binding afﬁnities [69]. However, the geometrical properties of the docking interface is invariant, regardless of the actual contribution of individual interactions therein. We argue that the class of toxin-channel interactions tested in this study emphasises surface matching, and HADDOCK can reliably produce “correct” binding modes that agree with mutagenesis data. The general success of *contact-pair* prediction implies that the native well is interfacially unique and clearly resolvable from the background of distant minima and non-speciﬁc binding. On the other hand, *afﬁnity* prediction fails by the virtue of frequent detection of false-positives and poor correlation of afﬁnity and HADDOCK energy. This implies that the accuracy of HADDOCK is insufﬁcient to accurately rank true binding. Such is the case for NTX and Pi1 where very similar modes have been replicated across the three channels. Of course, one should also note that arguments for selectivity can be made for toxins where surface compatibility is channel-speciﬁc, e.g. αKTx-3, HsTX1 and cobatoxin (not shown). Such geometrical aspects of binding should be discernable in docking through a lack of clustering and/or large changes in the binding mode location.

Can any hint of selectivity be observed? We note that electrostatics contribute to much of the binding process due to the net charges of the docking partners, and have previously observed such phenomena in PMF calculations [70,71]. The reaction intermediates in binding are dominated by electrostatics and show up in docking as a cloud over a broad set of coordinates. If it can be assumed that the absolute ’binding’ energy of these non-speciﬁc interactions are comparable across channels, then usable information on selectivity can be derived from the energetic separation of binding minima from the background.

**Figure 8:**
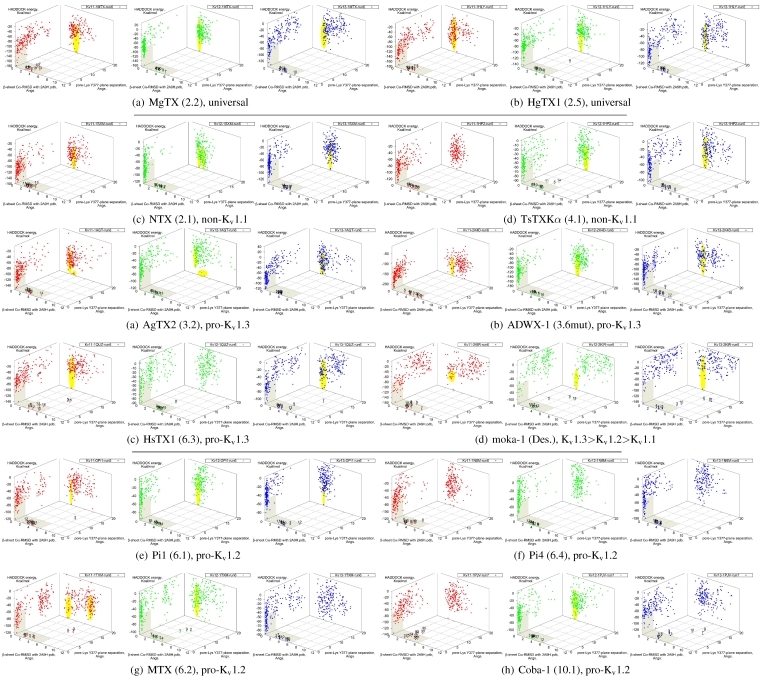
Constrained-docking data for twelve toxins from various families, grouped according to experimental phenotype. The colours red, greenand blue represent docking versus K_v_1.1, 1.2 and 1.3, respectively. The location of one or more major clusters that are potential models of the nativecomplex is additionally shaded yellow to aid interpretation. Its relative energy with repect to the non-binding background is potentially an indicatorof afﬁnity.

It is clear from the examples presented so far that most docking-pairs exhibit binding wells (of varying qualities) regardless of actual afﬁnity. This suggests that HADDOCK, being trained for binding site recognition, is energetically ineffective at distinguishing the false positives from the near-native complexes. On the other hand, a signiﬁcant portion of false complexes reveal deformations ranging from secondary structure of toxins to quaternary structure of channel assembly, although the scale can vary from minor distortions to gross deformations. We therefore attempted to improve minima determination by manually culling the un-physical complexes from the ﬁrst 30 complexes for a collection of toxins (Figure 8).

We ﬁnd that culling removes spurious outliers, selectively reduces cluster sizes, and improves binding-well shape marginally. While this assists with cluster identiﬁcation, the overall binding-well characteristics do not change signiﬁcantly. That is, this post-processing improves clustering characteristics but does not improve HADDOCK’s energetic discrimination. Using indicators of clustering, cluster-size and relative energy, a qualitative agreement with experimental afﬁnities for some toxins can be found: AgTX2 locations favour K_v_1.1 and 1.3 over K_v_1.2, with fracturing of clusters in K_v_1.2 and 1.3; TsTX-Kα–K_v_1.1 interactions show no distinctive clusters; HsTX1 and coba-1 show larger and more favoured clusters towards their target channels; mokatoxin (moka-1) displays the largest cluster *vs.* K_v_1.3. On the other hand NTX, Pi1 and MTX remain as before. Results for ubiquitous binders MgTX and HgTX1 are not predictive, but have been included to show their performance.

During culling we also noted that structural deformation tends to be more common in low-afﬁnity channel–toxin pairs. We suspect that the ultimate source of minima arises from the limitations of constraint-driven docking. The lack of membrane environments and artiﬁcial lowering of energetic barriers (to improve sampling efﬁciency) leads to artiﬁcial favouring of false minima relative to the mass of non-speciﬁc binding. In other words, during docking toxins are able to pay the costs of ﬁtting into sub-optimal binding orientations where realistic steric and conformational barriers would otherwise prevent this. This phenomenon can potentially be repaired by structure-quality checks (We did not utilise HADDOCK’s native interfaces with PROCHECK in this study) or additional reﬁnement stages where more realistic energy terms are utilised.

We also investigated the possibility that the inﬂuence of constraint-bias can be detected through blind-docking comparisons. This was able to identify NTX–K_v_1.1 as a false minima, as covered above—the removal of constraint bias drastically decreases the actual hit ratio of non-binders. For the rest of the toxins in this study, an approximate correlation between hit ratio and afﬁnity can sometimes be seen, especially for αKTx-3 toxins where the selectivity ratio is large. The methodology proved inconsistent, however, when we applied blind-docking checks to other families with more complex binding characteristics. The use of long MD-simulations to produce ensembles signiﬁcantly improves the comparative value of blind-docking, but did not eliminate the innate limitations of docking. Despite the increase of initial trials to 20,000 complexes to adequately sample the possible rotational arrangements, we cannot observe with conﬁdence whether lack of binding is due to non-interaction or lack of sampling of the binding-site. We can only conﬁrm whether our constraint choices accurately reﬂects the potential native modes that can exist for the toxin, *i.e.*, preservation of canonical binding. As clearly more sampling is required to make blind-docking statistically useful, we conclude that this method is not a cost-effective measure for systematic studies: blind-trials using 20,000 initial complexes already require ∼360 CPU-hours, as compared ∼96-CPU hours for a constrained trial with 5000 initial complexes.

### 3.8. Further Considerations and Extensibility of Protocol

In the interests of brevity and compatibility of interpretation, we elected to present only key toxins from the major αKTx-families—having selected only toxins that possess both experimental coordinates and afﬁnities towards rat K_v_-channels. Docking should in principle be applicable to other potassium channels if high-quality homology models can be built. Putative binders may then be identiﬁed by correlating functional residues across different decoys. The value of comparative docking lies in the explicit connection between sequence and function. For example, the αKTx-3 binding model is consistent across all tested members. This is a result that can be reinforced by building and testing additional members such as Aam-KTx (3.11) [72] and OdK2 (3.12) [73]. Assuming that protocols are sound, it should be expected that any member sharing sufﬁciently similar morphology and functional residues should yield comparative complexes. We would caution that all homology models, of toxins or channels, should be well-veriﬁed, e.g., tested for stability in long MD-simulations) before their application towards docking.

Our choice of pore-Lys as a measurement precludes us from making un-biased judgements of functional toxins that do not exhibit such a motif. It is evident in AgTX2-K27M that mutation can remove K^+^-ion competition without completely abrogating current-block [66], which echos similar alanine scan studies for other toxins. This supports the notion that other motifs are capable of supporting a pore-occluding mode. Aside from the central motif, the toxin-channel interface also contains numerous charge, hydrogen bond, and aromatic/sulphur interactions. It is therefore reasonable to suggest that current block can be sustained without a favourable pore interaction. On the other hand, it is possible that for some toxins a substitute from the basic-ring motif might form a low-afﬁnity mode. This presents a novel interface that afﬁrms some of the spurious false-positives found above, although such modes would remain in competition with K^+^-ions. Toxins from more recent families such as Tc32 (18.1) and Tt28(20.1) may be used to investigate these possibilities.

A generalisation of the “compare and contrast” protocol in this study requires the availability of a channel structure, a (small) collection of sequence-afﬁnity data from which to build a consensus, and some preliminary functional information to avoid a costly blind-search of the interaction surface. Although the correct binding mode can be resolved without assistance in the potassium channels investigated here, we suspect that this is not generally true due to the prominence of electrostatics within K_v_-binding. Given this consideration, adaptation to other potassium channels is a matter of homology-modelling and structural identiﬁcation of the toxins involved.

The recent publication of a bacterial voltage-gated sodium channel structure (NavAb, [74]) opens up the possibility of adapting this work towards human Na_v_ using corresponding selective peptides [75]. Of its pore-blocking toxins, analogous critical interactions such as E758–R13 in µ-conotoxin [76] may serve as a suitable constraint to increase computational efﬁciency. Structures for acid-sensing ion channels [77] also exist, although few toxins have been described thus far. A novel characterisation would involve a combination of mutagenesis scans with comparative docking, examining the perturbation of mutant on potential binding modes and clustering characteristics. We note that mutagenesis can also be used to artiﬁcially increase the “variety” of pairings.

We note that a signiﬁcant fraction of known channel toxins are gating modiﬁers that bind through voltage sensor domains. While a more complex deﬁnition of HADDOCK restraints can be constructed to describe the interface, extension of this protocol towards such peptides are complicated by membrane environment limitations. Nevertheless, in terms of selectivity between K_v_, Na_v_ and Ca_v_ voltage sensors both promiscuous and narrow toxins have been found. An investigation clarifying their structure-function relationships underlying this would be appropriate.

## 4. Summary

We ﬁnd that HADDOCK is adequate in identifying plausible mechanisms of binding across all of the αKTx families studied, using constrained-docking followed by analysis and clustering of docking performance to identify putative binding conﬁgurations. This process is somewhat complicated by the population of false minima in addition to true binding. Although the energy function reliably separates all putative minima from obvious non-binders, the separation of false from true minima is much more difﬁcult and will beneﬁt from some experimental knowledge of the interaction. While determination can be guided by close inspection of complex quality, such judgements will inevitably suffer from observer-bias. Especially in cases where the measureable binding to a K_v_-subtype is blocked only by one or two mutations, we ﬁnd parallel minima that closely resemble true binding against its active counterpart.

Despite these shortcomings, we can nevertheless draw predictions on familial characteristics with docking alone. HADDOCK preserves well the geometrical aspects of interactions, and by correlation across members of the same family we can link common selectivity proﬁle with common sequence features spotted in docking. The identiﬁcation of fundamental features also leads to analogies that can be extended to other families that possess convergent characteristics, and increases the conﬁdence in the interpretation of data from new toxins where validation is not yet available. With respect to detected familial characteristics, the αKTx-2 interact with conserved residues in the channel and αKTx-3 with the unique surface around the K_v_1.3 turret. The evidence for partial block in αKTx-6 and channel-dependent binding modes in αKTx-4 is tantalising, as HADDOCK gives testable models that can be investigated in experiments—the theorist must wait until such is carried out.

A general application of the ‘compare and contrast’ protocol in this study requires the availability of a channel structure, a (small) collection of sequence-afﬁnity data from which to build a consensus, and some preliminary functional information to avoid a costly blind-search of the interaction surface.

Although the information on contacts and categorisation of functional residues are helpful, HADDOCK is ultimately a qualitative tool. We have been unable to ﬁnd reliable correlations with experimental afﬁnity either by clustering or energetics. Some tendencies have been observed, and the fact that docking succeeds testiﬁes to an accurate reproduction of at least some properties in interaction. This is, however, not easily extractable from the present model. It remains to be seen whether improvements in the forceﬁeld model or intelligent re-weighting of contributors to the energy function can tease out these connections. Where quantitative information is direly needed, we suggest that further reﬁnements be made using more accurate computational methods, such as explicit free-energy calculations.

## Conﬂicts of Interest

The authors declare no conﬂict of interest.
